# Impact of change in maternal age composition on the incidence of Caesarean section and low birth weight: analysis of delivery records at a tertiary hospital in Tanzania, 1999–2005

**DOI:** 10.1186/1471-2393-9-30

**Published:** 2009-07-21

**Authors:** Projestine S Muganyizi, Hussein L Kidanto

**Affiliations:** 1 Department of Obstetrics and Gynecology, Muhimbili University of Health and Allied Sciences, Dar es Salaam, Tanzania; 2 Department of Obstetrics and Gynecology, Muhimbili National Hospital, Dar es Salaam, Tanzania

## Abstract

**Background:**

Previous studies on change in maternal age composition in Tanzania do not indicate its impact on adverse pregnancy outcomes. We sought to establish temporal changes in maternal age composition and their impact on annual Caesarean section (CS) and low birth weight deliveries (LBWT) at Muhimbili National Hospital in Tanzania.

**Methods:**

We conducted data analysis of 91,699 singleton deliveries that took place in the hospital between 1999 and 2005. The data were extracted from the obstetric data base. Annual proportions of individual age groups were calculated and their trends over the years studied. Multiple logistic analyses were conducted to ascertain trends in the risks of CS and LBWT. The impact of age composition changes on CS and LBWT was estimated by calculating annual numbers of these outcomes with and without the major changes in age composition, all others remaining equal. In all statistics, a p value < 0.05 was considered significant.

**Results:**

The proportion of teenage mothers (12–19 years) progressively decreased over time while that of 30–34 years age group increased. From 1999, the risk of Caesarean delivery increased steadily to a maximum in 2005 [adjusted OR = 1.7; 95%CI (1.6–1.8)] whereas that of LBWT declined to a minimum in 2005 (adjusted OR = 0.76; 95% CI (0.71–0.82). The current major changes in age trend were responsible for shifts in the number of CS of up to206 cases per year. Likewise, the shift in LBWT was up to 158 cases per year, but the 30–34 years age group had no impact on this.

**Conclusion:**

The population of mothers giving birth at MNH is progressively becoming older with substantial impact on the incidence of CS and LBWT. Further research is needed to estimate the health cost implications of this change.

## Background

Changes in age composition of the childbearing women population can strongly influence birth outcomes, health costs and social welfare of the women [1,2]. Such changes are reported in some countries, but the direction of change could differ between countries and even in the same country with time. Studies in the western countries generally report a decline in teen births [2-4] although the 40% rate of teenage pregnancy in USA has remained the highest [3]. Using hospital data in USA, the peak proportion of teenage mothers was recorded in the mid 1970s, but the proportion of older mothers steadily increased in late 1970s [5].

Studies in Africa indicate that overall Sub-Saharan Africa (SSA) has the highest rates of fertility in the world including that for adolescents. Since the 1980s, several countries in SSA have begun a transition toward lower fertility with an upward trend in the age at first birth, although wide variations still remain across countries and social groups [6].

There are many adverse pregnancy outcomes that are attributable to the extremes of maternal age. Advanced maternal age is associated with preterm birth [7-9], stillbirths [10-12] low birth weight and high CS rates [13-15]. Moreover, the risk for some important medical conditions and labor outcomes such as chronic hypertension, diabetes and blood loss is related to advanced maternal age [7]. Teenage, on the other hand, has been consistently associated with LBWT and anemia [13-15].

A number of factors have been put forward to explain changes in maternal age composition with time. Modernization, innovations in health care, increased education, and improved communications are some of the important factors linked with age composition changes among the childbearing population [16]. In Tanzania studies have indicated a substantial improvement in the average level of education for women, modernization and communication in the recent years [17,6]. It has also been shown that, the rate of giving birth before the age of 18 years has declined [6]. However, there is no study so far that has tried to link the major changes in maternal age composition with the burden of adverse pregnancy outcomes.

This study was focused on trends of maternal age at birth in order to identify changes in age composition for women who delivered at MNH between 1999 and 2005. Since such changes may give more weight to age groups at high risk for poor pregnancy outcomes, we also paid attention to the annual trends of CS and LBWT in order to establish association of their shifts to changes in age composition. This study aimed to answer three research questions:

1. Are there major temporal changes in age composition for mothers who delivered at MNH between 1999 and 2005?

2. Has the likelihood of adverse pregnancy outcomes (CS and delivery of LBWT) for mothers of variable age groups changed over the years?

3. What is the impact of the observed major changes in age composition to the annual burden of CS and LBWT over the years?

## Methods

### Study settings

Muhimbili National Hospital (MNH) is the largest consultant hospital in the United Republic of Tanzania being situated in Dar es Salaam, the country's largest city. According to the 2002 national population Census, the city has a total population of about 3.4 million with annual growth rate of 4.3%. The maternity unit is affiliated to the department of obstetrics and gynaecology in the MNH structure. The unit receives referred pregnant women from Dar es Salaam district hospitals as well as other hospitals from within the city. Occasionally it receives patients from other nearby regions. About 40 women deliver at this unit each day. The MNH also serves as teaching hospital for the Muhimbili University Health and Allied Sciences.

### The obstetric database

This study utilized information stored in the MNH electronic obstetric database whose details are given elsewhere [15]. The database was established in 1998 and data have been prospectively entered. Patients admitted to the MNH labor ward bring their antenatal cards and the information is entered into the admission book on arrival. Data on labor, maternal and neonatal outcomes are entered into the midwifery book. After delivery the information from the midwifery book is computerized. The obstetric database thus contains information on: maternal age, marital status, parity, APGAR scores at one and five minutes, birth weight, maternal and fetal outcomes as well as maternal complications. Validity of data entered in the database is ensured by a data quality program run weekly and validity checks of the data, done twice annually. The latter is done by annual comparison, between the information in the ledgers with the information, in the database for selected variables. From the electronic obstetric database we identified all singleton deliveries from 1999 to 2005 and analyzed their data.

### Data analysis

Data were captured by Epi info software and then exported to SPSS version 14 and Minitab-15 for analysis. Temporal changes in age were analyzed by calculating annual proportions made up by the various age groups among all singleton deliveries and plotted against years to produce graphical assessment of the trends. Chi square for linear trend was calculated to ascertain linearity for the important age groups.

Since multiple pregnancies constitute a risk for CS and LBWT such pregnancies were excluded in analysis. Multiple logistic regression was used to estimate and compare annual odds of CS deliveries and delivery of a LBWT over the years using 1999 as a reference. Then the regression was stratified by year using the two pregnancy outcome as dependent variables and age group as an independent categorical variable. The aim of the latter analysis was to establish whether apart from the change in age composition there was also a change in the risk of CS or LBWT for that particular age group. The 35–50 years age group was selected as a reference since it was known to be at high risk for CS and LBWT [15] and revealed a fairly stable trend over the years on a preliminary analysis of our results. In the regression analyses where delivery by CS was the binary outcome, we controlled for the confounding effects of Oxytocin use, delivery of LBWT, delivery of low APGAR score at 5 minutes, status of referral and parity. If LBWT was the binary outcome, we controlled for the status of referral and parity of the mother.

The impact of the major changes in age composition on CS delivery and LBWT was estimated by plotting shifts in the total annual number of CS and LBWT with or without the effect of the major age composition changes, all other factors remaining equal. For all statistics, a p value of 0.05 or less was considered significant. The study was ethically approved by the research and publications committee of the Muhimbili University of Health and allied sciences.

## Results

A total of 97,049 singleton deliveries took place at MNH between 1999 and 2005. Data for 5,350 (5.5%) deliveries were excluded because they were incomplete, thus remaining with 91,699 deliveries for analysis. Total annual deliveries progressively decreased from 15,595 in 1999 to 10,550 in 2005. However, annual CS deliveries increased from 2,354 (15.1%) in 1999 to 2,705 (25.6%) in 2005.

Characteristics of mothers and their deliveries are shown in Table 1. Maternal age ranged 12–50 years (mean = 25.36 ± 6.04) with more than half (57%) delivered at or between 20–29 years of age.

**Table 1:** Selected characteristics of mothers and singleton deliveries at MNH, 1999–2005

**Age at delivery(yrs)**	**Number**	**Percent**
12–19	16573	18
20–29	52385	57
30–34	14345	16
35–50	8396	9
**Status of referral**		
Non official	80705	88
Official	10994	12
**Parity**		
1	40187	44
2	23141	25
3–5	24006	26
6+	4365	5
**Mode of delivery**		
Vaginal	72431	79
CS	19268	21
**Birth Weight**		
2500 g or above	76889	84
1500 g–2499.9 g	11962	13
<1500 g	2848	3
**Use of Oxytocin**		
Not used	85198	93
Used	6501	7
**APGAR Score at five minutes**		
8–10	75095	82
≤ 7	16604	18

An overwhelming majority (88%) of the deliveries were by mothers whose admissions were made outside the official referral system (i.e., came directly from home). Overall 44% were first time births and the rate of LBWT delivery was 16%. The number and proportion of LBWT deliveries (i.e., birth weight <2500 g) decreased progressively from 2,732 (17.5%) in 1999 to 1,527 (14.5%) in 2005.

### Changes in maternal age and parity

**Figure 1:**
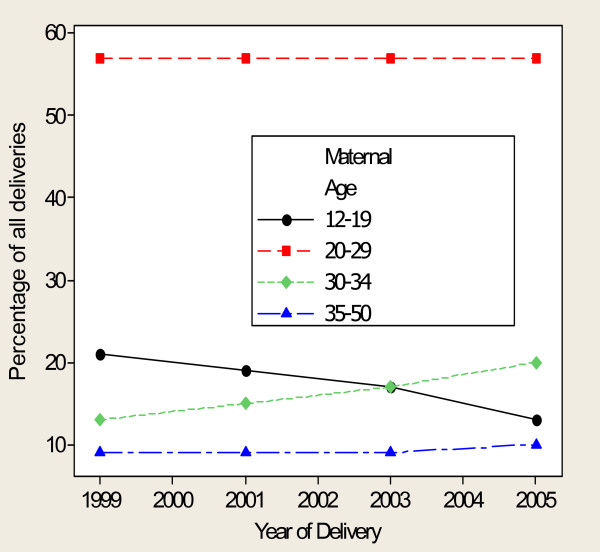
**Annual trends of age composition for mothers who delivered singleton pregnancies at MNH, 1999–2005**.

The annual proportion of teenage mothers declined linearly from 21% in 1999 to 13% in 2005, while that of mothers who were 30–34 years of age increased from 13% to 19% in the respective years (p < 0.001 each). The proportion of mothers delivering at ages 20–29 and 35–50 years demonstrated neither a consistent rise nor a decline over the years (Figure 1).

**Figure 2:**
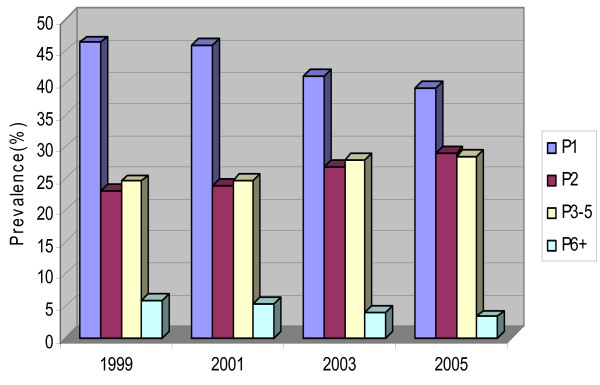
**Annual trends of Parity composition for mothers who delivered singleton pregnancies at MNH, 1999–2005**. P1 = Para1; P2 = Para 2; P3–5 = Para 3 to 5; P6+ = Para6 or above (Grand multipara).

The proportions of intermediate parity groups of mothers (i.e., Para 2, and Para 3–5) who delivered at the hospital had increased at the expense of the declining proportions of first time births and sixth time births or above. The proportion of mothers giving birth for the first time decreased from 46% in 1999 to 39% in 2005. A decrease for the grand-multiparous mothers (Para 6 or above) was from 6% in 1999 to 3% in 2005 (Figure 2).

### Risks for CS and LBWT

Comparing with what it was in 1999, the risk for CS steadily increased with time to a maximum in 2005 [adjusted OR = 1.7; 95%CI (1.6–1.8)]. In a stratified regression analysis by year of delivery and age group, throughout the study period, the risk for CS among teenage mothers remained persistently lower compared to the oldest age group (35–50 years) with the adjusted odds ratio ranging from 0.45 in 1999 to 0.49 in 2005. The odds of delivery by CS for the age group 30–34 years did not significantly change above or below that of the 35–50 years age group over the years [see additional file 1].

Overall the odds of delivery of LBWT babies steadily declined over years to reach the minimum in 2005 [adjusted OR = 0.76; 95% CI (0.71–0.82)]. Compared to 35–50 years age group after stratification, the adjusted odds of delivering a LBWT baby among teenage mothers declined from higher and significant in 1999 and 2001 to comparable risks in 2003 and 2005. For mothers aged 30–34 years, the risk remained comparable with that of the 35–50 years age group except for a fluctuation during 2001 when the risk was significantly higher for the 30–34 years age group [see additional file 2].

### Impact of age trends on CS and LBWT

**Figure 3:**
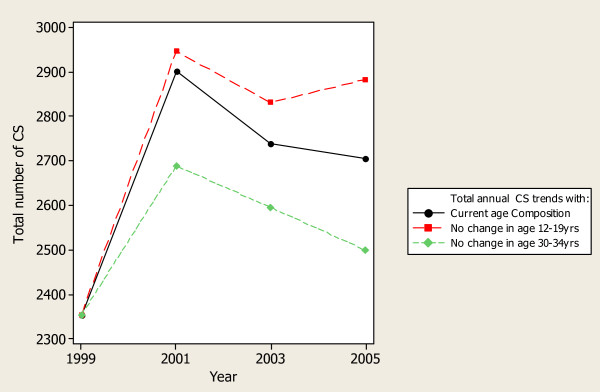
**Impact of the major changes in maternal age composition on annual CS deliveries at MNH, 1999–2005**.

**Figure 4:**
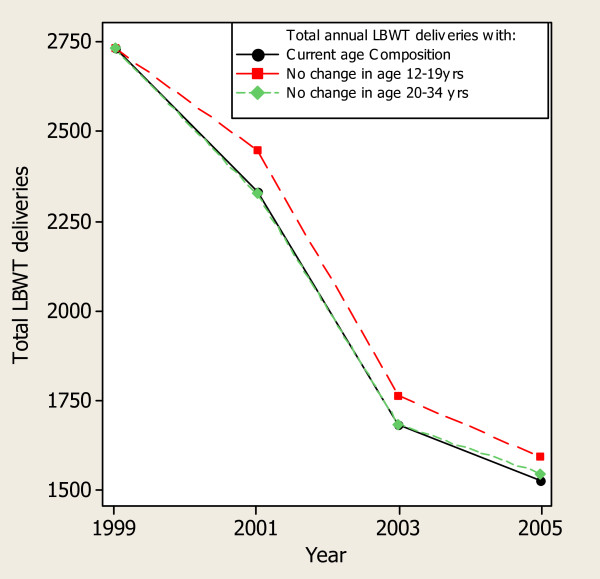
**Impact of the major changes in maternal age composition on annual LBWT deliveries at MNH, 1999–2005**.

The contribution of teenage mothers as a proportion of the total annual burden of CS decreased from 14% in 1999 to 10% in 2005. In contrast, the contribution to the burden of CS by the mothers of age 30–34 years increased from 15% to 23%. The impact of change in age composition is illustrated in figures 3&4. As can be seen in figure 3, if all other changes over the years (including the total annual number of deliveries and age specific CS rates) remained as they were currently, and the proportion of teenagers remained the same (21%) as it was in 1999, there would be more CS done year after year reaching the maximum in 2005 where 175 more CS would be performed. On the other hand, if the proportion of mothers delivering at the ages 30–34 yrs remained 13% in 2005 as it were in 1999, there would be 206 CS less in 2005.

As can be seen in Figure 4, the impact of change in age trends on delivery of LBWT was more marked for the 12–19 years age group. The maximum shift would be 158 more cases of LBWT in 2005. Changes in age group 30–34 years did not have remarkable impact on the number of LBWT deliveries. Notable, however, in both outcomes (CS and LBWT), shifts in the annual number attributable by the change in age composition did not bring back the number of the outcome events to the 1999-baseline level due to the combined impact of other factors.

## Discussion

This study has revealed a steady decline in the proportion of teenage mothers who delivered at MNH from 1999 to 2005, and a steady rise in the proportion of older (30–34 years) mothers. This shift in age composition coincides with a steady decline in the proportion of mothers who delivered for the first time and sixth time or more. When interpreted together, increasingly the population of mothers who delivered at MNH in the past decade has moved toward delay in childbirth and less number of deliveries. These findings are in support of a recent community survey data in Tanzania that indicate a declining tendency to deliver before the age of 18 years between 1991 and 1996 [6]. Moreover, this scenario is similar to the one usually reported in countries with sustained shift toward lower fertility [2-4,6]

The explanations for the change in age composition and fertility of the study population were outside the scope of this study. Although modernization and recent advances in women education level in Tanzania might have at least in part contributed to the decline in teenage deliveries; the role of modern family planning practice is most debatable. Recent data from Tanzania indicate a steady increase in the national average contraceptive use for mothers in the reproductive age from 6% in 1992 to 18% in 2005. This national average rate, however, is still one of the lowest in the world [17] and can not be expected to contribute substantially to the reported decline in fertility.

Furthermore, the rise in modern contraception use in Tanzania has not made any visible impact at a national level where the average fertility rate has remained 5.7 children per woman since 1996. However, this study was conducted in Dar es Salaam region where the average contraception use is over 30% for women in the reproductive age, which might partially explain the trends toward delivery at later ages and toward intermediate parities [17].

Condom use and postponement of sexual intercourse to later ages due to fear of HIV may be another possible explanation for the decline in teenage deliveries. Although increased abortion rates among adolescents is often a factor mentioned to account for the decline in adolescent pregnancies in the developed countries [4], there is no evidence to support this notion in Tanzania since the restrictive abortion law has not changed over the years. Although international data indicate a small increase in the incidence of unsafe abortion in Sub Saharan Africa between 2000 and 2003 [18], there is no evidence to suggest an appreciable increase in abortion rate among teenagers in Tanzania in recent years. Whatever is responsible for the shift in the age at birth, it must have been sustainable enough to cause another visible shift toward delivery at later ages. Currently this effect is visible with the 30–34 years group, but a rise involving the oldest group (35–50 years) can be anticipated in future as the result of a cohort effect if efforts to reduce fertility will not lead to the desired outcome.

The shift in age compositions toward delivery at older age can be associated with visible impact on pregnancy outcomes, neonatal and infant morbidity, mortality and health costs [4,5,14]. Our study used CS and LBWT deliveries as an example of important pregnancy outcomes. While it is established that the overall CS rate has increased at MNH over the study years [15], this study further revealed that the risk for delivery of LBWT has steadily declined during the same period.

Among the most important findings of this study is the impact of change in age composition on these two pregnancy outcomes (i.e., CS and LBWT). Our findings suggest that, the decline in teenage deliveries has substantially decreased the number of CS performed each year. In contrast, an increase in the proportion of older mothers (30–34 years) has overwhelmingly increased the number of CS deliveries each year.

Moreover, the contribution made by the change in age composition on the burden of CS does not seem to be related to preferential changes in the risk for CS for any of the two key age groups as compared to the reference after controlling for important confounders. This suggests that biological factors could be more important for the observed impact of change in age composition on CS [19].

Interestingly, the overall risk of delivering a LBWT at MNH declined steadily and substantially over the study years. The impact of change in age composition on LBWT, particularly for the teenage mothers was remarkable but not as much as for CS. Shifts in the annual number of LBWT deliveries would have been wider if it were not for the concomitant progressive decrease in the risk of LBWT compared to the reference age group. The reasons for the preferential fall in the risk of LBWT were not established by this study but changes in the social-economical status of the study population could be responsible albeit in part. Decreased maternal body mass index, life style toward reduced smoking, reduced alcohol intake, improved education, and being married may all reduce the risk of LBWT [20,21].

Apart from social factors, obstetric factors like previous pregnancy outcomes and timing of conception may contribute significantly to delivery of LBWT [1,4]. The current database at MNH does not produce reliable information on the gestation age and detailed previous obstetric history variables. However, decreased rates of premature deliveries can be expected as the delivering population gets older. In contrast to studies elsewhere that have associated the rise in LBWT deliveries with the rise in CS rate [5], in the current study setting LBWT has declined despite the increased CS rates, indicating that the association of the two can not be generalized to all settings.

Although our data were derived from records in a tertiary hospital, the study population would not be expected to differ substantially from that of the women who deliver at lower health facilities in Tanzania given that ninety percent of them came directly from home without being referred. This fact is further reinforced by similar results regarding teenage deliveries from a previous community survey in Tanzania [6]. While the situation may be closer to what is taking place at the lower health facilities, our study population is in no way representing the general community. In addition to this limitation, the use of retrospective data did not permit us to obtain all the variables we needed for the analysis. Particularly socio-economical factors were missed, while they would be important confounders of our study outcomes.

This study was conducted at the time of increased global campaign on healthy timing and spacing of pregnancy based on recent studies about the risks of adverse perinatal outcomes [10-15,18-22] and modernization [16]. As a consequence of the global campaign and modernization, further changes in age composition of the childbearing women population can be anticipated particularly in the developing countries. The rise in CS and the decrease in LBWT attributable to changes in maternal age composition suggest the reproductive health importance of temporal changes in demographic and possibly social composition of the maternal population which is rarely addressed by clinicians and managers in our study setting. Since such changes can have important implications on health costs and maternal and fetal health outcomes, future research should lead to more understanding of these dynamics and the consequences they have on reproductive health at community and health facility levels in Tanzania.

## Conclusion

This study has revealed a steady decline in the proportion of teenage and a rise in the proportion of older (30–34 years) mothers. These changes in age composition have contributed substantially to the parallel increase in CS rate and the decrease in LBWT deliveries at MNH. Future studies should establish other contributing factors to the trends of these pregnancy outcomes and analyze the cost implications of the change in age composition.

## Competing interests

The authors declare that they have no competing interests.

## Authors' contributions

PSM participated in the concept development, design of the study, data cleaning, statistical analysis, interpretation of the data, writing the first draft of the manuscript and approval of the final draft.

HLK participated in data collection, statistical analysis, interpretation of the data, development of the final draft and approval of the manuscript

## Pre-publication history

The pre-publication history for this paper can be accessed here:



## Supplementary Material

Additional file 1 **Table 2: Adjusted risks for CS at MNH by year of delivery and maternal age**. The table represents data on adjusted odds ratios and 95% confidence intervals for CS delivery at MNH from 1999 to 2005 for mothers of different age groups. Click here for file

Additional file 2 **Table 3: Adjusted risk for LBWT at MNH by year of delivery and maternal age**. The table represents data on adjusted odds ratios and 95% confidence intervals for LBWT delivery at MNH from 1999 to 2005 for mothers of different age groups. Click here for file
