# Supplementary material for: Impact of change in maternal age composition on the incidence of Caesarean section and low birth weight: analysis of delivery records at a tertiary hospital in Tanzania, 1999–2005
Source: BMC Pregnancy Childbirth. 2009 Jul 21;9:30. doi: 10.1186/1471-2393-9-30 (PMC2718860; doi:10.1186/1471-2393-9-30)
Supplement: Additional file 1 — Table 2: Adjusted risks for CS at MNH by year of delivery and maternal age. The table represents data on adjusted odds ratios and 95% confidence intervals for CS delivery at MNH from 1999 to 2005 for mothers of different age groups. [file 1471-2393-9-30-S1.doc]

**Table 2:** Adjusted risks for CS at MNH by year of delivery and maternal age

|  | **1999** | | **2001** | | **2003** | | **2005** | |
| --- | --- | --- | --- | --- | --- | --- | --- | --- |
|  | **All**  **deliveries** | **Adjusted^*^**  **OR(95% CI)** | **All**  **deliveries** | **Adjusted^*^**  **OR(95% CI)** | **All**  **deliveries** | **Adjusted^*^**  **OR(95% CI)** | **All**  **deliveries** | **Adjusted^*^**  **OR(95% CI)** |
| **Age group(yrs)** |  |  |  |  |  |  |  |  |
| 12-19 | 3332 | 0.45(0.36,0.57) | 2751 | 0.48(0.39,0.60) | 1873 | 0.47(0.38,0.59) | 1317 | 0.49(0.39,0.61) |
| 20-29 | 8869 | 0.76(0.63,0.93) | 8222 | 0.78(0.65,0.93) | 6278 | 0.69(0.57,0.82) | 6039 | 0.68(0.58,0.80) |
| 30-34 | 2012 | 0.91(0.75,1.1) | 2091 | 1.2(1.00,1.5) | 1912 | 0.97(0.80,1.2) | 2048 | 0.95(0.80,1.1) |
| 35-50 | 1382 | 1.00 (referent) | 1317 | 1.00 (referent) | 981 | 1.00 (referent) | 1091 | 1.00(referent) |

*Adjusted for Oxytocin use, delivery of LBWT, delivery of low APGAR score at 5 minutes, status of referral and parity
