# Supplementary material for: Impact of change in maternal age composition on the incidence of Caesarean section and low birth weight: analysis of delivery records at a tertiary hospital in Tanzania, 1999–2005
Source: BMC Pregnancy Childbirth. 2009 Jul 21;9:30. doi: 10.1186/1471-2393-9-30 (PMC2718860; doi:10.1186/1471-2393-9-30)
Supplement: Additional file 2 — Table 3: Adjusted risk for LBWT at MNH by year of delivery and maternal age. The table represents data on adjusted odds ratios and 95% confidence intervals for LBWT delivery at MNH from 1999 to 2005 for mothers of different age groups. [file 1471-2393-9-30-S2.doc]

**Table 3: Adjusted risk for LBWT at MNH by** year of delivery and maternal age

|  | **1999** | | **2001** | | **2003** | | **2005** | |
| --- | --- | --- | --- | --- | --- | --- | --- | --- |
|  | **All**  **deliveries** | **Adjusted^*^**  **OR(95% CI)** | **All**  **deliveries** | **Adjusted^*^**  **OR(95% CI)** | **All**  **deliveries** | **Adjusted^*^**  **OR(95% CI)** | **All**  **deliveries** | **Adjusted^*^**  **OR(95% CI)** |
| **Age group(yrs)** |  |  |  |  |  |  |  |  |
| 12-19 | 3332 | 1.4(1.1,1.8) | 2751 | 1.6(1.2,2.0) | 1873 | 1.1(0.82,1.4) | 1317 | 0.98(0.76,1.3) |
| 20-29 | 8869 | 1.1(0.89,1.3) | 8222 | 1.2(1.0,1.5) | 6278 | 0.88(0.71,1.1) | 6039 | 0.82(0.66,1.0) |
| 30-34 | 2012 | 1.1(0.87,1.3) | 2091 | 1.3(1.1,1.7) | 1912 | 1.00(0.83,1.3) | 2048 | 0.94(0.76,1.2) |
| 35-50 | 1382 | 1.00 (referent) | 1317 | 1.00 (referent) | 981 | 1.00 (referent) | 1091 | 1.00(referent) |

*Adjusted for status of referral and parity
